# Application of the international criteria for optic neuritis in the Acute Optic Neuritis Network

**DOI:** 10.1002/acn3.52166

**Published:** 2024-08-04

**Authors:** Philipp Klyscz, Susanna Asseyer, Ricardo Alonso, Charlotte Bereuter, Omer Bialer, Atira Bick, Sara Carta, John J. Chen, Leila Cohen, Yamit Cohen‐Tayar, Edgar Carnero Contentti, Russell C. Dale, Eoin P. Flanagan, Jonathan A. Gernert, Julian Haas, Joachim Havla, Christoph Heesen, Mark Hellmann, Netta Levin, Pablo Lopez, Itay Lotan, Maria Belen Luis, Sara Mariotto, Christina Mayer, Alvaro Jose Mejia Vergara, Cassandra Ocampo, Susana Ochoa, Frederike C. Oertel, Maja Olszewska, José Luis Peralta Uribe, Jaume Sastre‐Garriga, Dario Scocco, Sudarshini Ramanathan, Natthapon Rattanathamsakul, Fu‐Dong Shi, Jemal Shifa, Ilya Simantov, Sasitorn Siritho, Alon Tiosano, Nanthaya Tisavipat, Isabel Torres, Adi Vaknin Dembinsky, Angela Vidal‐Jordana, Adi Wilf‐Yarkoni, Ti Wu, Sol Zamir, Luis Alfonso Zarco, Hanna G. Zimmermann, Axel Petzold, Friedemann Paul, Hadas Stiebel‐Kalish

**Affiliations:** ^1^ Experimental and Clinical Research Center A Cooperation between Max Delbrück Center for Molecular Medicine in the Helmholtz Association and Charité ‐ Universitätsmedizin Berlin Berlin Germany; ^2^ Department of Neurology, Charité ‐ Universitätsmedizin Berlin Corporate Member of Freie Universität Berlin and Humboldt‐Universität zu Berlin Berlin Germany; ^3^ Max Delbrück Center for Molecular Medicine in the Helmholtz Association (MDC) Berlin Germany; ^4^ Neuroscience Clinical Research Center (NCRC), Charité ‐ Universitätsmedizin Berlin Corporate Member of Freie Universität Berlin and Humboldt‐Universität zu Berlin Berlin Germany; ^5^ University Center of MS and NMOSD, Neurology Department Ramos Mejia Hospital Buenos Aires Argentina; ^6^ Department of Neuro‐Ophthalmology Rabin Medical Center Petah Tikva Israel; ^7^ Sackler Faculty of Medicine Tel Aviv University Tel Aviv Israel; ^8^ Department of Neurology, Hadassah Medical Center Hebrew University Jerusalem Israel; ^9^ Neurology Unit, Department of Neurosciences, Biomedicine, and Movement Sciences University of Verona Verona Italy; ^10^ Department of Neurology Mayo Clinic Rochester Minnesota USA; ^11^ Center for MS and Autoimmune Neurology Mayo Clinic Rochester Minnesota USA; ^12^ Department of Ophthalmology Mayo Clinic Rochester Minnesota USA; ^13^ Eye Laboratory, Felsenstein Medical Research Center Tel Aviv University Tel Aviv Israel; ^14^ Neuroimmunology Unit, Department of Neuroscience Hospital Aleman Buenos Aires Argentina; ^15^ TY Nelson Department of Paediatric Neurology Children's Hospital at Westmead Sydney New South Wales Australia; ^16^ Faculty of Medicine and Health and Brain and Mind Centre University of Sydney Sydney New South Wales Australia; ^17^ Clinical Neuroimmunology Group, Kids Neuroscience Centre Children's Hospital at Westmead Sydney New South Wales Australia; ^18^ Department of Laboratory Medicine and Pathology Mayo Clinic Rochester Minnesota USA; ^19^ Institute of Clinical Neuroimmunology, LMU Hospital Ludwig‐Maximilians‐Universität Munich Munich Germany; ^20^ Institute of Neuroimmunology and Multiple Sclerosis University Medical Center Hamburg Eppendorf Hamburg Germany; ^21^ Neuroimmunology Service, Department of Neurology Rabin Medical Center Petah Tikva Israel; ^22^ Neuromyelitis Optica Research Laboratory, Massachusetts General Hospital Harvard Medical School Boston Massachusetts USA; ^23^ Ophthalmology Department University of Florida Gainesville Florida USA; ^24^ Faculty of Medicine University of Botswana Gaborone Botswana; ^25^ Pontificia Universidad Javeriana and Hospital Universitario San Ignacio Bogotá Colombia; ^26^ Neurology Department, Multiple Sclerosis Centre of Catalonia (Cemcat) Vall d’Hebron University Hospital Barcelona Spain; ^27^ Department of Neurology Concord Hospital Sydney New South Wales Australia; ^28^ Translational Neuroimmunology Group, Kids Neuroscience Centre Children's Hospital at Westmead Sydney New South Wales Australia; ^29^ Faculty of Medicine Siriraj Hospital Mahidol University Bangkok Bangkok Thailand; ^30^ Department of Neurology Tianjin Medical University General Hospital Tianjin China; ^31^ Department of Surgery University of Botswana Gaborone Botswana; ^32^ Neuroscience Center Bumrungrad International Hospital Bangkok Thailand; ^33^ Department of Neurology, Sackler Faculty of Medicine Tel Aviv University Tel Aviv Israel; ^34^ Einstein Center Digital Future Berlin Germany; ^35^ The National Hospital for Neurology and Neurosurgery University College London London UK; ^36^ Moorfields Eye Hospital London UK; ^37^ Neuro‐ophthalmology Expert Centre Amsterdam Netherlands

## Abstract

**Objective:**

The first international consensus criteria for optic neuritis (ICON) were published in 2022. We applied these criteria to a prospective, global observational study of acute optic neuritis (ON).

**Methods:**

We included 160 patients with a first‐ever acute ON suggestive of a demyelinating CNS disease from the Acute Optic Neuritis Network (ACON). We applied the 2022 ICON to all participants and subsequently adjusted the ICON by replacing a missing relative afferent pupillary defect (RAPD) or dyschromatopsia if magnetic resonance imaging pathology of the optical nerve plus optical coherence tomography abnormalities or certain biomarkers are present.

**Results:**

According to the 2022 ICON, 80 (50%) patients were classified as definite ON, 12 (7%) patients were classified as possible ON, and 68 (43%) as not ON (NON). The main reasons for classification as NON were absent RAPD (52 patients, 76%) or dyschromatopsia (49 patients, 72%). Distribution of underlying ON etiologies was as follows: 78 (49%) patients had a single isolated ON, 41 (26%) patients were diagnosed with multiple sclerosis, 25 (16%) patients with myelin oligodendrocyte glycoprotein antibody‐associated disease, and 15 (9%) with neuromyelitis optica spectrum disorder. The application of the adjusted ON criteria yielded a higher proportion of patients classified as ON (126 patients, 79%).

**Interpretation:**

According to the 2022 ICON, almost half of the included patients in ACON did not fulfill the requirements for classification of definite or possible ON, particularly due to missing RAPD and dyschromatopsia. Thorough RAPD examination and formal color vision testing are critical to the application of the 2022 ICON.

## Introduction

Optic neuritis (ON) describes an inflammation of the optic nerve that is a common manifestation of demyelinating autoimmune diseases such as multiple sclerosis (MS), aquaporin‐4‐IgG positive (AQP4‐IgG+) and seronegative neuromyelitis optica spectrum disorder (NMOSD) and myelin oligodendrocyte glycoprotein (MOG) antibody‐associated diseases (MOGAD).^1^, ^2^ Worldwide incidence of ON is between 1 and 3 per 100.000 person per year.^3^ The prevalence of various etiologies of ON differs around the globe with a higher percentage of antibody‐mediated ON in patients of Asian or African descent.^3^, ^4^ Furthermore, ON can be caused by other diseases such as infections or systemic disorders. Therefore, the correct diagnosis of ON is crucial for acute treatment as well as long‐term therapeutic decisions and prognostications.^2^

Optic neuritis typically manifests by visual loss, dyschromatopsia, and visual field defects. Visual symptoms are often preceded by ipsilateral orbital pain increasing with eye movements.^1^ Clinical examination usually shows a relative afferent pupillary defect (RAPD), while fundoscopy might reveal an optic disc swelling.^5^ The diagnostic work‐up in patients with suspected ON is aimed at ruling out infectious, granulomatous, rheumatological disorders, and in appropriate settings also investigations of paraneoplastic causes.^6^ Additional test includes magnetic resonance imaging (MRI) of the brain and optic nerves, optical coherence tomography (OCT), cerebrospinal fluid (CSF) analysis, and serum antibody testing for AQP4‐IgG and MOG‐IgG and may include visually evoked potentials.^2^, ^7^, ^8^ Typical optic neuritis is a clinical diagnosis, which can be made based on the medical history and ophthalmological findings alone, especially as advanced diagnosis tools are not equally accessible in all areas of the world.

Prior studies reported high rates of misdiagnosis in patients with suspected ON.^9^ To address this issue, the 2022 International consensus criteria for ON (ICON) by Petzold et al., defined by a Delphi process‐based approach, were published recently.^6^ The criteria aimed to increase the certainty of ON diagnosis in clinical practice and future trials by incorporating certain clinical and paraclinical aspects. So far, the 2022 ICON was only assessed in retrospective studies and thus lack a general applicability. The aim of this work is to (1) prospectively apply the 2022 ICON to all consecutive patients recruited in the ongoing, prospective Acute Optic Neuritis Network (ACON)^10^ and (2) to evaluate the performance of an exploratory adjustment of the 2022 ICON compared to the original criteria.

## Methods

### Study population

Subjects included in ACON between August 2020 and December 2023, in an ongoing, multicenter, prospective observational trial, investigating the effect of time‐to‐treatment in acute ON, were evaluated.

As described in detail elsewhere,^10^ patients are eligible to participate in ACON if they present with a first‐ever acute ON (i.e., single isolated optic neuritis [SION], MS‐ON, NMOSD‐ON, or MOGAD‐ON) evaluated within 30 days of symptom onset. Optic neuritis diagnosis was made based on best clinical practice. Exclusion criteria comprised of preexisting demyelinating disease and any other form of optic neuropathy (e.g., hereditary, ischemic, infectious, granulomatous). Currently, ACON comprises 40 academic institutions and tertiary hospitals in Africa, Asia, Australia, Europe, and the Middle East, North, and South America. For the current analysis, patients from the following countries were included: Argentina, Australia, Botswana, China, Colombia, Germany, Israel, Italy, Spain, Thailand, and the USA.

Demographic data such as sex, age, ethnicity, final diagnosis, and therapy were collected. For the current analysis, baseline and follow‐up data, if available, were used. The diagnosis of MS was based on McDonald 2017 criteria^11^, the diagnosis of NMOSD was based on the International Panel for NMO Diagnosis (IPND) 2015 criteria,^12^ and diagnosis of MOGAD was done according to the International MOGAD Panel proposed criteria^13^. Single isolated optic neuritis was defined as idiopathic ON if they did not meet the criteria for MS, NMOSD, or MOGAD.

The clinical evaluation consisted of medical history, neurological assessment according to the Expanded Disability Status Scale (EDSS), RAPD testing, high‐contrast visual acuity testing, OCT, MRI, and serological analysis if available. Imaging data analyses were reported at the discretion of each center. Study data were stored and managed using Research Electronic Data Capture hosted at Charité Universitätsmedizin Berlin, Germany.^14^

### Application of the 2022 international diagnostic criteria for ON

Each center was responsible for applying the 2022 ICON to the clinical and paraclinical findings of their enrolled patients in ACON.^6^ The criteria allow a diagnosis of definite, possible, or no ON (NON) based on the following clinical and paraclinical features.

Clinical features of the 2022 ICON consist of

- A: Monocular, subacute loss of vision associated with orbital pain worsening on eye movements, reduced contrast and color vision, and relative afferent pupillary deficit.
- B: Painless with all other features of (A).
- C: Binocular loss of vision with all features of (A) or (B).

Paraclinical criteria consist of

- MRI: abnormalities (i.e., contrast enhancement or intrinsic signal increase of the affected optic nerve acutely) of the optic nerve consistent with ON within 3 months of onset.
- OCT: optic disc swelling acutely or significant intereye difference (defined as a macular ganglion and inner plexiform layer (mGCIPL) of >4% or >4 μm or in the peripapillary retinal nerve fiber layer (pRNFL) of >5% or >5 μm) within 3 months.
- Biomarker: detection of AQP4‐IgG, MOG‐IgG, or collapsin response mediator protein 5 (CRMP5)‐antibody in serum or the presence of oligoclonal bands (OCB) in the CSF.

Patients were classified as definite ON when

- clinical features of A and one additional paraclinical test,
- clinical features of B and two different paraclinical tests, or
- clinical features of C and two different paraclinical tests of which one was an abnormal MRI were present.

According to the 2022 ICON, diagnosis of possible ON was made when clinical characteristics of either A, B, or C descriptions (i.e., vision loss, dyschromatopsia, and RAPD) were present but paraclinical requirements for definite ON were incomplete. Cases of monocular vision loss with orbital pain, but in whom RAPD was deemed absent and in whom dyschromatopsia was undocumented, were classified as NON according to the 2022 ICON.

### Statistical analysis

Demographic data, ON etiology, and clinical features at disease onset were compared between subjects with different ON classifications. For continuous variables, means and standard deviations (SD) were reported and group comparisons were conducted using a Kruskal–Wallis test or independent t test. For categorical variables, absolute numbers and percentages were reported and comparisons were conducted using either chi‐square test (if applicable with Yates' continuity correction) or the Fisher' exact test.

To ensure that the group of patients with definite ON/probable ON was comparable with the group classified as NON, we performed the following additional analysis for each group:

1. Time from reported vision loss onset to date when visual acuity at nadir was documented,
2. Time from initiation of acute therapy with corticosteroids to date on which visual acuity at nadir was tested, and
3. Nadir visual acuity.

In patients classified as NON according to the 2022 ICON, adjusted criteria were applied and defined as follows: Patients who were initially classified as NON due to missing RAPD and/or dyschromatopsia but had an abnormal MRI scan consistent with acute ON as described in the 2022 ICON definitions^6^ and an additional positive paraclinical test were reclassified as ON. Because a distinguishment between possible and definite ON, in this case, is obsolete, we dichotomized the original classification by subsuming cases with definite and possible ON for better comparison.

Statistical analyses were performed using R (version 4.2.2). The *p*‐values of <0.05 were defined as statistically significant.

### Standard protocol approvals, registrations, and patient consents

The institutional review board approval was obtained from Charité—Universitätsmedizin Berlin as well as from all participating centers as part of ACON. Written informed consent was obtained from all participants. The ACON study is registered at clinicaltrials.gov (No. NCT05605951) and performed in accordance with the Declaration of Helsinki.

## Results

In total, 171 patients are currently included in ACON. Three patients were excluded due to screening failure (two with a history of previous ON and one with retinal sarcoidosis) and eight patients due to missing data. Therefore, 160 patients were included in the final analysis. Characteristics of all included patients, separated for each diagnosis are summarized in Table 1. Among the 160 subjects, 68% were female, 68% were of white ethnicity, and the mean age was 37 years (SD 12 years). Patients with NMOSD‐ON were older (age [mean ± SD]: 42 ± 14 years) and predominantly female (93%).

**Table 1:** Summary of patient characteristics by ON etiologies.

	Overall *N* = 160	SION *N* = 78	MS‐ON *N* = 41	NMOSD‐ON *N* = 15	MOGAD‐ON *N* = 26
Sex, female (present, *n* (%))	109 (68%)	50 (64%)	28 (68%)	14 (93%)	17 (65%)
Age in years (mean, SD)	37 (12)	39 (13)	33 (8)	43 (14)	36 (10)
Ethnicity (present, *n* (%))					
White	109 (68%)	53 (68%)	33 (80%)	5 (33%)	18 (69%)
Latin	16 (10%)	9 (12%)	1 (2.4%)	3 (20%)	3 (12%)
Arab	11 (7%)	7 (9%)	0 (0%)	1 (7%)	3 (12%)
Asian	9 (6%)	7 (9.0%)	0 (0%)	0 (0%)	2 (8%)
Black	5 (3%)	0 (0%)	3 (7%)	2 (13%)	0 (0%)
HCVA at nadir in decimals (mean, SD)	0.35 (0.35)	0.39 (0.35)	0.50 (0.36)	0.09 (0.20)	0.16 (0.23)
Unilateral painful vision loss (present, *n* (%))	109 (68%)	58 (74%)	31 (76%)	8 (53%)	12 (46%)
Unilateral painless vision loss (present, *n* (%))	21 (13%)	10 (13%)	9 (22%)	0 (0%)	2 (8%)
Bilateral vision loss (present, *n* (%))	30 (19%)	10 (13%)	1 (2.4%)	7 (47%)	12 (46%)
Dyschromatopsia (present, *n* (%))	111 (69%)	59 (76%)	25 (61%)	11 (73%)	16 (62%)
RAPD (present, *n* (%))	108 (68%)	57 (73%)	26 (63%)	11 (73%)	14 (54%)
OCT abnormalities (present, *n* (%))^a^	70 (44%)	36 (46%)	17 (41%)	6 (40%)	11 (42%)
MRI abnormalities (present, *n* (%))^a^	128 (80%)	57 (73%)	32 (78%)	14 (93%)	25 (96%)
Biomarker (OCB, AQP4‐, MOG‐IgG) present (present, *n* (%))	81 (51%)	16 (21%)	28 (68%)	14 (93%)	26 (100%)

AQP4‐IgG, aquaporin‐4‐IgG; MOG‐IgG, myelin oligodendrocyte glycoprotein‐IgG; MOGAD, myelin oligodendrocyte glycoprotein antibody‐associated disease; MRI, magnetic resonance imaging; MS, multiple sclerosis; NMOSD, neuromyelitis optica spectrum disorder; OCB, oligoclonal bands; OCT, optical coherence tomography; ON, optic neuritis; RAPD, relative afferent pupillary defect; SD, standard deviation; SION, single isolated optic neuritis.

^a^ According to abnormalities described in 2022 ICON definitions.^6^

After diagnostic work‐up, most patients had a SION (78, 49%), followed by MS‐ON (41, 26%), MOGAD‐ON (26, 16%), and NMOSD‐ON (15, 9%). Most patients with NMOSD‐ON were positive for AQP4‐IgG (14/15 patients, 93%). With regards to clinical features, most patients experienced monocular vision loss with painful eye movements (SION 74%, MS‐ON 76%, NMOSD‐ON 53%, and MOGAD‐ON 46%). Bilateral vision loss was most frequent in patients with NMOSD‐ON (47%) and MOGAD‐ON (46%). Monocular painless vision loss was infrequent across all etiologies (SION 13%, MS‐ON 22%, MOGAD‐ON 8%, and NMOSD‐ON 0%). High‐contrast visual acuity was more severely affected in NMOSD‐ON and MOGAD‐ON (0.09 [SD 0.20] and 0.16 [SD 0.23], respectively), followed by SION (0.39 [SD 0.35]) and MS‐ON (0.50 [SD 0.36]).

**Figure 1:**
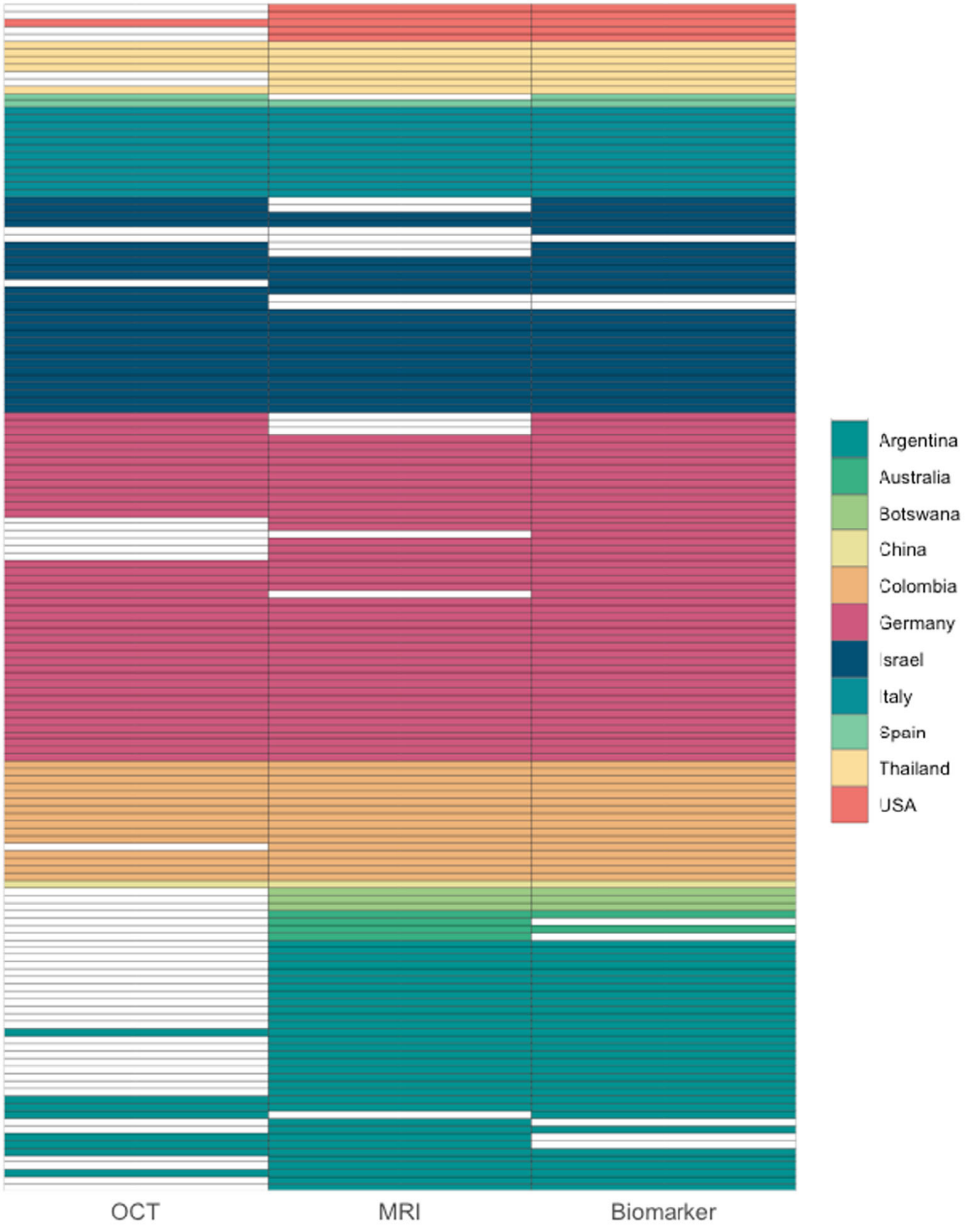
Availability of OCT, MRI, and biomarker (AQP4‐/MOG‐IgG, OCB) testing for all patients. Each row represents an individual patient. If any diagnostic procedure is not available, the respective tile is left blank. OCT was the least available paraclinical test in our cohort. AQP4‐IgG, aquaporin‐4‐IgG; MOG‐IgG, myelin oligodendrocyte glycoprotein‐IgG; MRI, magnetic resonance imaging; OCB, oligoclonal bands; OCT, optical coherence tomography.

The availability of diagnostic procedures for each patient is depicted in Figure 1. Most patients received biomarker testing (146 patients, 91%) and an MRI (124 patients, 78%). Optical coherence tomography was least frequently performed particularly in Argentinian centers and available in 97 patients (61%).

After the application of the 2022 ICON, 80 (50%) patients were classified as definite ON, 12 (8%) as possible ON, and 68 (42%) as NON (Table 2). Seventy‐three patients (91%) classified as definite ON had gadolinium enhancement or increased intrinsic signal of the affected optic nerve in MRI, 49 (61%) patients tested positive for CSF‐restricted OCB, serum AQP4‐IgG, or MOG‐IgG, and 46 (58%) patients had an optic disc swelling or a reported intereye difference of >4% or >4 μm in the GCIPL or of >5% or >5 μm in the pRNFL (thinning or swelling), respectively. In patients classified as possible ON according to 2022 ICON, four patients (33%) had MRI abnormalities of the optic nerve, four patients (33%) had OCT abnormalities consistent with ON, and two patients (17%) tested positive for one of the prespecified biomarkers.

**Table 2:** Characteristics of patients separated by 2022 ICON classification.

	Overall *N* = 160	Definite ON *N* = 80	Possible ON *N* = 12	NON *N* = 68
Sex, female (present, *n* (%))	109 (68%)	51 (64%)	8 (67%)	50 (74%)
Age in years (mean, SD)	37 (12)	37 (12)	43 (13)	36 (10)
ON etiologies (present, *n* (%))				
SION	78 (49%)	39 (49%)	9 (75%)	30 (44%)
MS‐ON	41 (26%)	21 (26%)	1 (8.3%)	19 (28%)
NMOSD‐ON	15 (9.4%)	9 (11%)	1 (8.3%)	5 (7.4%)
MOG‐ON	26 (16%)	11 (14%)	1 (8.3%)	14 (21%)
Ethnicity (present, *n* (%))				
White	109 (68%)	50 (61%)	9 (75%)	50 (74%)
Latin	16 (10%)	12 (15%)	0 (0%)	4 (6%)
Arab	11 (7%)	6 (8%)	1 (8%)	4 (6%)
Asian	9 (6%)	6 (8%)	2 (3%)	1 (8%)
Black	5 (3%)	1 (1%)	0 (0%)	4 (6%)
HCVA in decimals (mean, SD)	0.35 (0.35)	0.31 (0.34)	0.48 (0.40)	0.39 (0.35)
Dyschromatopsia (present, *n* (%))	111 (69%)	80 (100%)	12 (100%)	19 (28%)
RAPD (present, *n* (%))	108 (68%)	80 (100%)	12 (100%)	16 (24%)
OCT abnormalities (present, *n* (%))^a^	70 (44%)	46 (58%)	4 (33%)	20 (29%)
MRI abnormalities (present, *n* (%))^a^	128 (80%)	73 (91%)	4 (33%)	51 (75%)
Biomarker (OCB, AQP4‐, MOG‐IgG) (present, *n* (%))	81 (51%)	49 (61%)	2 (17%)	33 (49%)

AQP4‐IgG, aquaporin‐4‐IgG; MOG‐IgG, myelin oligodendrocyte glycoprotein‐IgG; MOGAD, myelin oligodendrocyte glycoprotein antibody‐associated disease; MS, multiple sclerosis; MRI, magnetic resonance imaging; NMOSD, neuromyelitis optica spectrum disorder; OCB, oligoclonal bands; OCT, optical coherence tomography; RAPD, relative afferent pupillary defect; SD, standard deviation; SION, single isolated optic neuritis.

^a^ According to abnormalities described in 2022 ICON definitions.^6^

**Figure 2:**
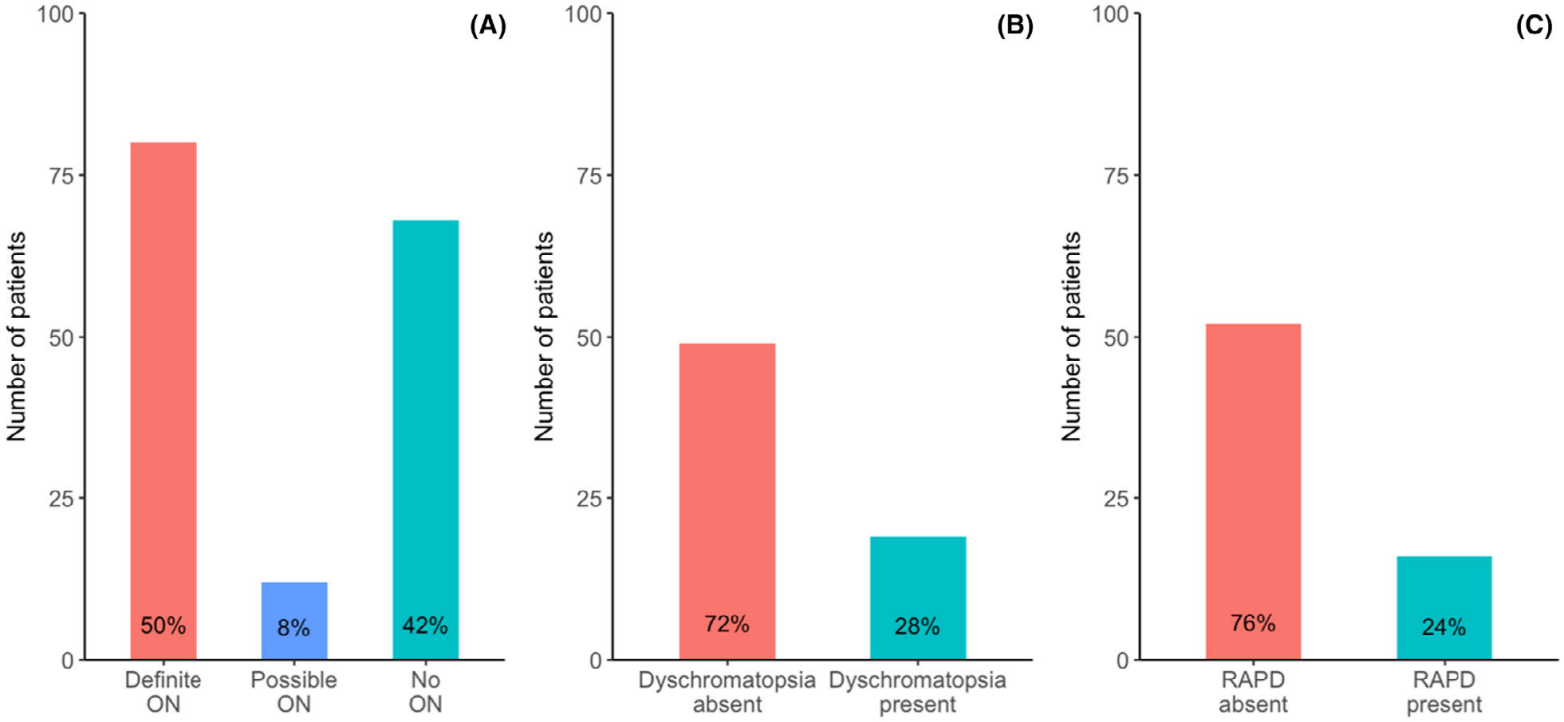
Bar plots showing distribution among 160 ACON patients meeting the 2022 ICON (A), the number of patients with documented dyschromatopsia (B), and the percentage of patients in whom RAPD was documented (C). ACON, Acute Optic Neuritis Network; ICON, international consensus criteria for optic neuritis; ON, optic neuritis; RAPD, relative afferent pupillary defect.

Out of 65 patients classified as NON, 47 (72%) did not have reported dyschromatopsia and 50 (77%) did not have a confirmed RAPD (Fig. 2). However, among the 65 NON patients, 41 (63%) had MRI abnormalities consistent with ON, 28 (43%) had a positive biomarker suggestive of demyelinating disease, and 15 (23%) had ON compatible pathologies in OCT imaging (Table 2).

Patient characteristics across the entire cohort did not differ with respect to the 2022 ICON classification results (*p*‐value for sex 0.440, *p*‐value for ethnicity 0.421, and *p*‐value for ON etiologies 0.507). Moreover, the comparison between patients classified as definite or probable ON versus those patients classified as NON according to the 2022 ICON did not yield a significant difference in (1) the time from vision loss to visual acuity measurement (mean in ON 6.1 days, mean in NON 7.7 days, *p* = 0.473), (2) the time from initiation of corticosteroid therapy to nadir visual acuity documentation (mean in ON 1.1 days, mean in NON 1.9 days, *p* = 0.510), or (3) the nadir high‐contrast visual acuity (mean in ON 0.32, mean in NON 0.39, *p* = 0.250).

The relative afferent pupillary defect was present in 73% of painful monocular ON and 81% of painless monocular ON, while in bilateral ON, only 34% had an evident RAPD (*p* = 0.000). Dyschromatopsia was also least frequent in bilateral ON (60%) compared to painful (72%) and painless (67%) unilateral ON. However, this difference was not statistically significant (*p* = 0.405). Also, paraclinical abnormalities were comparable between ON phenotypes (*p*‐value for abnormal OCT pathologies 0.192, *p*‐value for abnormal MRI 0.624, *p*‐value for biomarker presence 0.623).

**Figure 3:**
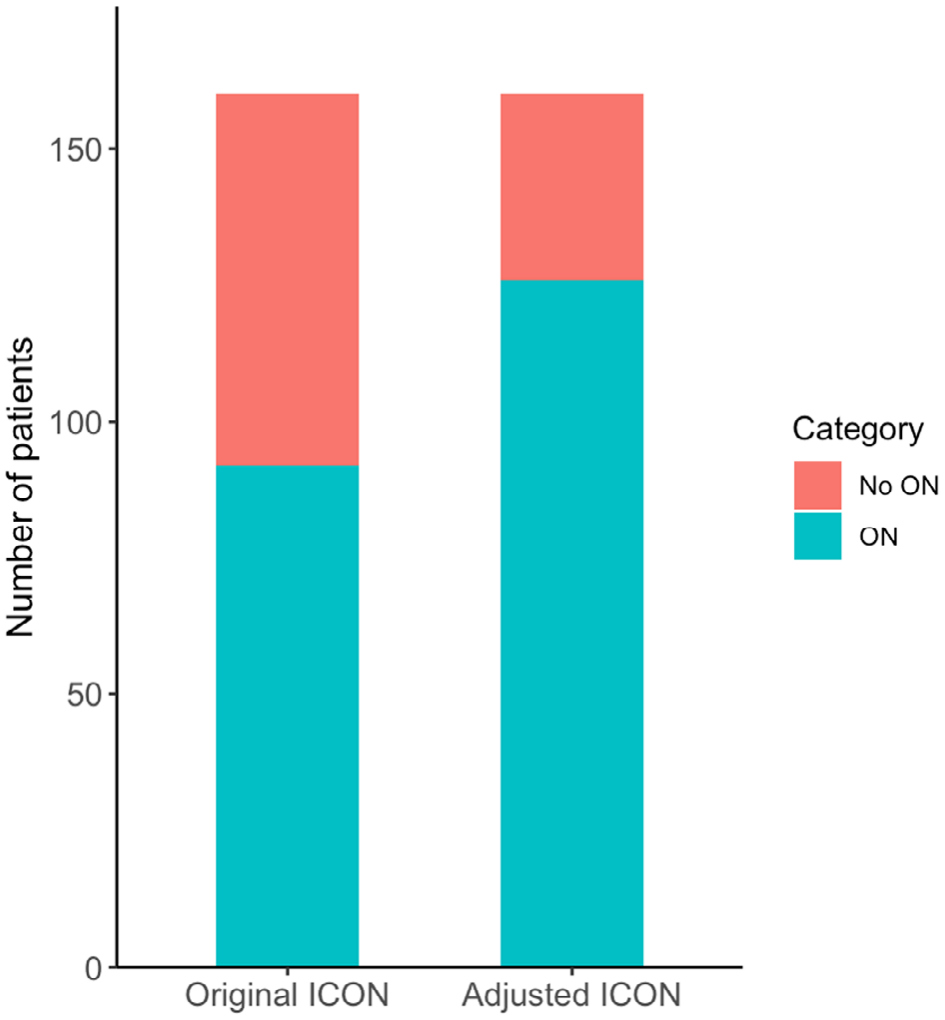
Comparative bar plot shows that substituting clinical symptoms with paraclinical signs of the 2022 ICON results in a higher proportion of patients classified as ON. First, original ON criteria were dichotomized by joining definite and possible ON. For the adjusted criteria, all patients that were initially classified as NON (due to missing RAPD or dyschromatopsia), were reclassified as ON if they had MRI pathology of the affected optic nerve and were positive for at least one additional paraclinical criteria (i.e., OCT pathology or presence of AQP‐/MOG‐IgG/OCB). AQP4‐IgG, aquaporin 4‐IgG; ICON, international consensus criteria for optic neuritis; MOG‐IgG, myelin oligodendrocyte glycoprotein‐IgG; MRI, magnetic resonance imaging; NON, no ON; OCB, oligoclonal bands; OCT, optical coherence tomography; ON, optic neuritis; RAPD, relative afferent pupillary defect.

Finally, we applied the modified criteria to the entire cohort (Fig. 3). After dichotomization of the original 2022 ICON, 92 (58%) patients were classified as ON and 68 patients (42%) as NON. The adjustment of the criteria (i.e., replacement of clinical symptoms through positive paraclinical tests) resulted in a higher proportion of patients classified as ON (126 patients, 79%).

## Discussion

In this cross‐sectional analysis of patients included in the prospective, multicenter cohort study, ACON, we found that only half of the subjects were classified as definite ON according to the 2022 novel ICON. The main reasons for not fulfilling the criteria were absent RAPD or dyschromatopsia on clinical examination.

Making a correct diagnosis of ON remains challenging. Until the publication of the 2022 ICON, no definitions for ON have been generally accepted.^6^ So far, most clinical characteristics as well as therapeutic strategies of ON were derived from the North America Optic Neuritis Treatment Trial (ONTT)^15^, a landmark study from 1992 that investigated the effect of corticosteroids on visual outcomes in ON. However, the ONTT focused on unilateral ON and was conducted before the availability of antibody testing and OCTs. Another important aspect to consider is that ON etiologies vary geographically. Even though ON seems to have a higher incidence in Western countries, the proportion of antibody‐mediated ON is higher in African, Asian, and Latin‐American countries.^4^, ^5^ Furthermore, nondemyelinating ON such as systemic diseases (e.g., sarcoidosis) and infections (e.g., toxoplasmosis, cat‐scratch disease) must be excluded in atypical cases and considered differential diagnoses in the evaluation of all patients.^16^ Therefore, the authors of the 2022 ICON had to consider multiple aspects to assure high diagnostic accuracy and prevent misdiagnosis. A retrospective analysis by Stunkel et al. revealed a high proportion of misdiagnosis of ON, particularly due to overreliance on a solitary medical history finding or neglect of alternative diagnosis.^9^ This might lead to unnecessary invasive diagnostic procedures (i.e., lumbar punctures) and inappropriate medical treatments including immunosuppression.

The 2022 ICON focused on three core clinical characteristics of ON: pain, dyschromatopsia, and RAPD.^6^ ON‐related orbital pain with worsening eye movements is very common in inflammatory demyelinating etiologies of ON. In MS‐ON and NMOSD‐ON, pain is usually mild to moderate and usually precedes visual loss.^17^ Particularly in MOGAD‐ON, eye pain can be very severe as the perineural tissue is frequently affected.^18^, ^19^ The location and severity of ON‐related pain might not only depend on the ON etiology. Epidemiological studies examining the clinical characteristics of ON in different ethnicities showed lower proportions of orbital pain as well as differences in localization and extent of ON in patients from Japan.^20^

Dyschromatopsia is a form of impaired color vision that may be congenital or acquired. In the 2022 ICON, no preferential tool for dyschromatopsia testing was specified. Desaturation of the color red is frequently used as a rapid substitute for established color vision tests in the diagnostic work‐up of a patient with suspected ON in an emergency room setting.^21^ There does not seem to be a difference in dyschromatopsia rates between MS‐ON, NMOSD‐ON, and MOGAD‐ON.^2^

The relative afferent pupillary defect is a clinical sign in the affected ON eye revealed through the swinging flashlight test demonstrated by a sluggish pupil response to direct illumination. It represents an impairment of the afferent limb of the pupillary reflex through affection of the retinal ganglion cell pathway and is a sign of asymmetric visual input from one eye, which is usually due to an optic neuropathy but can also be caused by a retinal process.^6^ Symmetric bilateral ON will not have an RAPD.

So far, only a few peer‐reviewed publications regarding the retrospective application of the 2022 ICON has been published.^22^, ^23^, ^24^ Terrim et al. investigated real‐world data from 77 patients with an inflammatory ON of a single‐center cohort and determined how many were possible or definite ON according to 2022 ICON. Most of the included subjects were classified as definite ON (62%). MS‐ON and SION were the most frequent etiologies of ON in this cohort. Similar to our cohort, the proportion of patients with unilateral painful ON was 72.5%. Nevertheless, the analysis by Terrim et al. and our current examination can only partially be compared, as (1) they did not include information on ON classified as NON according to the 2022 ICON, (2) they required confirmation by MRI of the optic nerve lesion, and (3) even ON in subjects without RAPD have been classified as possible or definite ON. Alvarez et al. evaluated the performance of the 2022 ICON in patients with noninflammatory optic neuropathies. They found an overall excellent performance of the 2022 ICON with a misdiagnosis rate of 2%.^24^

Further, a letter exchange on the 2022 ICON by Gingele et al.^25^ pointed out that in their retrospective validation study, the RAPD was absent in 83% and dyschromatopsia absent in 62% of the patients with ON.^25^ In a subsequent retrospective study of the same group by Jendretzky et al., 59% of included patients did not fulfill the 2022 ICON for definitive ON. Likewise, the main reason for the classification of NON in this cohort was missing RAPD (72%) or dyschromatopsia (57%).^23^ However, the authors did not discuss the reasons for the high rates of missing RAPD and dyschromatopsia in their cohort. They proposed that missing core clinical features should also be substituted through paraclinical tests.^23^

One may consider that neither the timing of screening for these core clinical characteristics nor examination skills were specified in the 2022 ICON. Patients presenting with painful vision loss are often screened by general ophthalmologists in the setting of busy emergency departments. These patients are subsequently referred to neurologists and corticosteroid treatment may have been initiated before extended screening for dyschromatopsia, RAPD, and pain were obtained.

Unlike orbital pain, RAPD and color vision testing, cannot be replaced by paraclinical tests in the 2022 ICON. When we applied the novel diagnostic criteria to the ACON cohort, we found that 42% did not fulfill the requirements for classification of a definite or possible ON, mostly due to missing RAPD or color vision impairment. The RAPD was tested for each case, as assessment of the 2022 ICON is a part of patient screening for recruitment to the ACON study. However, RAPD could possibly be very subtle and difficult to detect in a patient with only mild unilateral ON and preserved vision^26^ or if testing of the RAPD is performed after significant recovery. Moreover, detection of a slight RAPD is dependent on the examiner's experience, an optimal setting for the clinical examination (i.e., a very dark room that might not be available in the setting of an emergency room screening or using a light source that is not bright enough), and a good distance fixation on behalf of the patient. RAPD may not be present in patients with previous ON of the contralateral eye or in patients with bilateral ON. As we included only patients with inaugural ON, we can mostly rule out prior ON as a possible cause of the lack of RAPD. Additionally, in patients with bilateral asymmetric ON, observing an RAPD can be challenging in clinical routine. This observation is supported by our data, as RAPD was least frequently detected in patients with bilateral ON. In a retrospective, longitudinal study from Finland, a similar number of patients compared to the ACON cohort had an RAPD (92 of 121 patients (76%)).^27^ Generally, a missing RAPD should raise concerns regarding the veracity of the diagnosis of ON.^28^ We suggest in patients with probable ON but questionable/no RAPD, obtaining a second RAPD evaluation by a neuro‐ophthalmologist for confirmation would be helpful. However, if the patient has had significant improvement prior to the RAPD evaluation, its utility and sensitivity will be lower.

Data regarding the rates of dyschromatopsia among patients with ON are sparse. Most epidemiological data regarding dyschromatopsia is derived from the ONTT. In the ONTT, ON patients with an RAPD present in the ophthalmologic examination were included. At baseline, 93% of the included patients had impaired color vision which persisted in 41% after 6 months.^29^ The ONTT tested dyschromatopsia with validated color vision testing procedures, including both the Farnsworth‐Munsell‐100‐Hue test and the Ishihara pseudoisochromatic plates.^30^ Further studies in patients with chronic ON have verified a long‐term impairment of color vision and showed an association with retinal structural parameters.^31^ Apart from the ONTT‐derived data, several studies with small sample sizes have also verified the presence of dyschromatopsia in acute ON.^32^, ^33^, ^34^

We adjusted the 2022 ICON by adding the possibility to replace RAPD and dyschromatopsia through paraclinical tests consisting of MRI abnormalities of the affected optic nerve and an additional pathological paraclinical test of a different modality, including abnormalities in OCT or the presence of specific biomarkers. Our goal was to raise the precision of the novel ON criteria. Thereby, we were able to classify patients as ON which would have been classified as NON according to the original 2022 ICON because RAPD or dyschromatopsia was absent even though anamnesis and paraclinical test clearly suggested an ON diagnosis. This might offer the possibility in the future to correctly diagnose patients with ON with only very subtle clinical abnormalities or inconclusive RAPD testing.

Making a diagnosis of ON as early as possible is crucial to elevate the chances of amelioration of visual impairment.^35^, ^36^ This might be particularly important in patients with NMOSD‐ON and MOGAD‐ON, where visual acuity can be severely impaired.^2^ According to the 2022 ICON, five patients with NMOSD‐ON and 14 patients with MOGAD‐ON from our cohort were classified as NON. After applying the adjusted ON criteria, only one patient with NMOSD‐ON was still classified as NON due to missing MRI enhancement of the optic nerve. Therefore, medical personnel should be careful to discard a possible diagnosis of ON too early based on the 2022 ICON.

The strengths of the current analysis are that we applied the 2022 ICON to a global, multiethnic cohort, thus representing patients from different parts of the world. To our knowledge, this is the only prospective validation of the 2022 ICON as well as presently the largest analysis in terms of patient number.

Nevertheless, several limitations need to be discussed. As per protocol, we included only patients with an inaugural ON according to local standard clinical practice. Therefore, it was not possible to carry out a sensitivity or specificity analysis of the 2022 ICON. Consequently, we could not test for the positive or negative predictive value. As no other consented diagnostic criteria for ON have been published beforehand, we had no comparison regarding the performance of the 2022 ICON. A relatively high proportion of patients did not have an RAPD at presentation. We assumed this might be partially explained by the inclusion of bilateral ON and a large number of ON patients with only mild vision loss, in whom a subtle RAPD can be difficult to capture. Furthermore, patients who were diagnosed with MS‐ON might have had a prior subclinical ON in the contralateral eye, potentially masking an RAPD.^37^ In these cases, RAPD can be easily missed. A large number of cases with absent dyschromatopsia can be attributed to missing quantitative color vision testing in routine emergency department screening. Most centers in ACON assessed color vision by medical history rather than using established color vision tests (such as Ishihara or Hardy Rand Rittler tests). Therefore, we expect that dyschromatopsia was underdiagnosed. Further, regarding the adjusted ICON criteria, some patients did not receive a T1 fat‐suppressed contrast‐enhanced orbital imaging sequence which might have decreased MRI sensitivity in a very small number of patients. Last, the presence of CRMP5 antibodies was not recorded. CRMP5‐IgG is a very rare paraneoplastic antibody that might cause a variety of neurologic symptoms, typically in the context of small‐cell lung cancer. As we did not include patients with cancer, and given the rapid progression of small‐cell lung cancer, the likelihood of overlooking CRMP5‐ON seems very remote indeed. In addition, CRMP5‐ON tends to cause papillitis rather than the classic painful optic neuritis seen in inflammatory demyelinating diseases.^2^

In conclusion, this is the largest prospective validation of the 2022 ICON. We showed that the classification of ON is highly dependent on color vision testing, and a skilled evaluation of the relative afferent pupillary reflex. When we amended the 2022 ICON, we could slightly improve its performance. Therefore, we suggest a potential addendum to the 2022 ICON: patients with painful monocular vision loss in whom RAPD or dyschromatopsia were not documented at presentation, be included as ON if two additional diagnostic paraclinical criteria with one being MRI optic nerve abnormalities^6^ are present. These adjustments could be discussed in future iterations of the 2022 ICON.

## Conflict of Interest

PK, RA, CB, OB, AB, SC, LC, YC‐T, JH, MH, NL, PL, IL, MBL, CM, AJMV, CO, SO, MO, JLPU, JS‐G, DS, NR, F‐DS, JS, IS, SS, AT, NT, IT, AVD, AW‐Y, TW, SZ, and LAZ did not report any disclosures. SA received speaker's honoraria from Bayer, Alexion, Roche, and research grants from Stiftung Charité, Fritz‐Thyssen‐Stiftung, HEAD Genuit Stiftung, Rahel Hirsch Program, Novartis, and Roche. JJC is a consultant to UCB and Horizon, unrelated to this study. ECC has received reimbursement for developing educational presentations, educational and research grants, consultation fees and/or travel stipends from Biogen Argentina y LATAM, Genzyme Argentina, Merck Argentina y LATAM, Roche Argentina y LATAM, Raffo, Novartis Argentina, MERZ Argentina, Biosidus, Astrazeneca Argentina, Horizon Global, Amgen Argentina, the Guthy‐Jackson Charitable Foundation (Los Angeles, CA, USA), The Sumaira Foundation (Boston, MA, USA) and LACTRIMS. RCD received research funding from the Star Scientific Foundation, The Trish Multiple Sclerosis Research Foundation, Multiple Sclerosis Research Australia, the Petre Foundation, and the NHMRC (Australia; Investigator Grant). He has also received honoraria from Biogen Idec as an invited speaker and is on the IDMC for a Roche RCT in pediatric MS. He is on the medical advisory board (nonremunerated position). EPF served on advisory boards for Alexion, Genentech, Horizon Therapeutics, and UCB. He has received research support from UCB. He has received speaker honoraria from Pharmacy Times. He received royalties from UpToDate. He is a site principal investigator in a randomized clinical trial of Rozanolixizumab for relapsing myelin oligodendrocyte glycoprotein antibody‐associated disease run by UCB. He is a site principal investigator and a member of the steering committee for a clinical trial of satralizumab for relapsing myelin oligodendrocyte glycoprotein antibody‐associated disease run by Roche/Genentech. He has received funding from the NIH (R01NS113828). Dr Flanagan is a member of the medical advisory board of the MOG project. He is an editorial board member of Neurology, Neuroimmunology and Neuroinflammation, The Journal of the Neurological Sciences and Neuroimmunology Reports. A patent has been submitted on DACH1‐IgG as a biomarker of paraneoplastic autoimmunity. JAG reports travel expenses and nonfinancial support from Merck, outside the submitted work. JH reports grants from Friedrich‐Baur‐Stiftung, Merck, and Horizon; personal fees and nonfinancial support from Alexion, Horizon, Roche, Merck, Novartis, Biogen, B.M.S., and Janssen; and nonfinancial support from the Guthy‐Jackson Charitable Foundation and The Sumaira Foundation. CH received research grants from Merck, Novartis, and speaker honoraria from Merck and Roche. SM received speakers' honoraria from Alexion, Novartis, Biogen, Sanofi, and Horizon unrelated to this study. FCO currently receives research funding from the Hertie Foundation for Excellence in Clinical Neurosciences and Novartis, both unrelated to this project. She also received fellowship support by the American Academy of Neurology (until 2023) and the National Multiple Sclerosis Society (until 2023), both unrelated to this project.

JS‐G serves as a co‐editor for Europe for the *Multiple Sclerosis Journal* and as an Editor‐in‐Chief of *Revista de Neurología*, receives research support from Fondo de Investigaciones Sanitarias (19/950 and 22/750), and in the last, 12 months has served as a consultant/speaker for BMS, Roche, Sanofi, Janssen, and Merck. SR received research funding from the National Health and Medical Research Council (NHMRC, Australia), the Petre Foundation, the Brain Foundation, the Royal Australasian College of Physicians, and the University of Sydney. She is supported by an NHMRC Investigator Grant (GNT2008339). She serves as a consultant on an advisory board for UCB and Limbic Neurology and has been an invited speaker for educational/research sessions coordinated by Biogen, Alexion, Novartis, Excemed, and Limbic Neurology. She is on the medical advisory board (nonremunerated positions) of The MOG Project and the Sumaira Foundation. AV‐J has received support from contracts Juan Rodes (JR16/00024) and from Fondo de Investigación en Salud (PI17/02162 and PI22/01589) from Instituto de Salud Carlos III, Spain, and in the last 2 years, she has engaged in consulting and/or participated as speaker in events organized by Roche, Novartis, Merck, and Sanofi. HGZ reports research grants and speaker honoraria from Novartis. AP reports personal fees from Novartis, Heidelberg Engineering, and Zeiss, grants from Novartis, outside the submitted work; and AP is part of the steering committee of the OCTiMS study which is sponsored by Novartis. AP is part of the steering committee of Angio‐OCT which is sponsored by Zeiss. He does not receive honorary as part of these activities. The NIHR BRC at Moorfields Eye Hospital supported AP. FP served on the scientific advisory boards of Novartis and MedImmune; received travel funding and/or speaker honoraria from Bayer, Novartis, Biogen, Teva, Sanofi‐Aventis/Genzyme, Merck Serono, Alexion, Chugai, MedImmune, and Shire; is an associate editor of Neurology: Neuroimmunology & Neuroinflammation; is an academic editor of PLoS ONE; consulted for Sanofi Genzyme, Biogen, MedImmune, Shire, and Alexion; received research support from Bayer, Novartis, Biogen, Teva, Sanofi‐Aventis/Geynzme, Alexion, and Merck Serono; and received research support from the German Research Council, Werth Stiftung of the City of Cologne, German Ministry of Education and Research, Arthur Arnstein Stiftung Berlin, EU FP7 Framework Program, Arthur Arnstein Foundation Berlin, Guthy‐Jackson Charitable Foundation, and NMSS. HS‐K reports consulting fees and nonfinancial support from Roche, and Quark, consulting fees from the Israel Ministry of Health, and research grants from the Maratier Foundation for Vision Research and the Israel Motor Vehicle accident‐prevention authority, unrelated to this study.

## Author Contributions

PK, SA, and HSK conceptualized the study, wrote the first draft, and were responsible for data acquisition, analysis, methodology, literature review, and project administration. All other authors were responsible for data collection and patient recruitment at their centers, gave critical input to the manuscript, supported the literature research, and helped editing the paper.

## Supporting information


Data S1.

## Data Availability

The data supporting the findings in the present study will be made available upon reasonable request.
