# Supplementary material for: Application of the international criteria for optic neuritis in the Acute Optic Neuritis Network
Source: Ann Clin Transl Neurol. 2024 Aug 4;11(9):2473–84. doi: 10.1002/acn3.52166 (PMC11537134; doi:10.1002/acn3.52166)
Supplement: Supplementary file 1 — Data S1. [file ACN3-11-2473-s001.docx]

**Supplemental Material**

Application of the international criteria for optic neuritis in the Acute Optic Neuritis Network

Supplemental figure 1. Overview of included patients from each center.


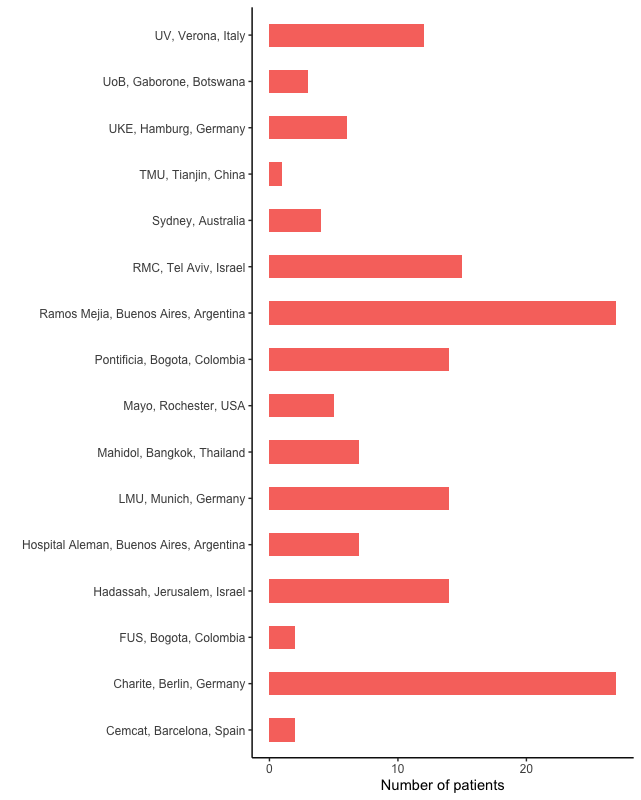


Abbreviations: Cemcat, Centre d’Esclerosi Múltiple de Catalunya; FUS, Fundación Universitaria Sanitas; LMU, Ludwig-Maximilians-Universität; RMC, Rabin Medical Center; TMU, Tianjin Medical University; UKE, Universitätsklinikum Hamburg-Eppendorf; UoB, University of Botswana; UV, University of Verona

Supplemental table 1. Results of CSF analysis across the entire cohort.

|  | **Overall**  N = 160 | **SION**  N = 78 | **MS-ON**  N = 41 | **NMOSD-ON**  N = 15 | **MOGAD-ON**  N = 26 |
| --- | --- | --- | --- | --- | --- |
| White blood cell count in cells/μl (mean, SD) | 7.4 (15.6) | 4.2 (4.1) | 10.3 (12.4) | 4.9 (4.7) | 12.2 (31.4) |
| Total protein in mg/dl (mean, SD) | 74 (129) | 75 (110) | 99 (180) | 36 (36) | 51 (104) |
| Oligoclonal bands in CSF (present, n (%)) | 49 (42%) | 16 (30%) | 29 (91%) | 1 (10%) | 3 (13%) |

Abbreviations: CSF, cerebrospinal fluid; MOGAD, myelin oligodendrocyte glycoprotein antibody-associated disease; MS, multiple sclerosis; NMOSD, neuromyelitis optica spectrum disease; OCB, oligoclonal bands; SD, standard deviation; SION, single isolated optic neuritis
